# Characterization of Fungal Foams from Edible Mushrooms Using Different Agricultural Wastes as Substrates for Packaging Material

**DOI:** 10.3390/polym15040873

**Published:** 2023-02-10

**Authors:** Nur Mawaddah Majib, Sung Ting Sam, Noorulnajwa Diyana Yaacob, Nor Munirah Rohaizad, Wai Kian Tan

**Affiliations:** 1 Faculty of Chemical Engineering & Technology, Universiti Malaysia Perlis (UniMAP), Arau 02600, Perlis, Malaysia; 2 Centre of Excellence Geopolymer and Green Technology (CEGeoGTech), Universiti Malaysia Perlis (UniMAP), Arau 02600, Perlis, Malaysia; 3 Institute of Nano Electronic Engineering, Universiti Malaysia Perlis, Seriab, Kangar 01000, Perlis, Malaysia; 4 Institute of Liberal Arts and Sciences, Toyohashi University of Technology, Toyohashi 441-8580, Aichi, Japan

**Keywords:** mycelium, agriculture wastes, fungal foam, oyster mushroom, lignocellulosic

## Abstract

Agricultural wastes and leaves, which are classified as lignocellulosic biomass, have been used as substrates in the production of fungal foams due to the significant growth of the mushroom industry in recent years. Foam derived from fungi can be utilized in a variety of industrial applications, including the production of packaging materials. Here, white oyster mushrooms (*Pleurotus florida*) and yellow oyster mushrooms (*Pleurotus citrinopileatus*) were cultivated on rice husk, sawdust, sugarcane bagasse, and teak leaves. Fungal foams were produced after 30 days of incubation, which were then analyzed using scanning electron microscopy (SEM), thermal analysis (TGA), and chemical structure using Fourier-transform infrared spectroscopy. Mechanical testing examined the material’s hardness, resilience, and springiness, and water absorption tests were used to determine the durability of the fungal foams. Our findings demonstrated that fungal foams made from rice husk and teak leaves in both mycelium species showed better mechanical properties, thermal stability, and minimal water absorption compared to the other substrates, and can thus have great potential as efficient packaging materials.

## 1. Introduction

Waste production is increasing as a result of the expanding population, rapid industrialization, and urbanization. Consequently, many innovative processes and solutions have been developed, implemented, and improved upon to reduce the negative impact of waste on society and the ecosystem. Until now, biodegradable waste has been a valuable resource in various applications such as composting or as a resource for heat and electricity, through the recycling process. However, due to inadequate or in some cases nonexistent recycling procedures, the global economy has yet to reach a closed-loop materials cycle.

Although approximately 280 million tons of plastic are manufactured every year, only 10% is actually recycled [1]. Considering that plastic waste pollution has caused significant environmental issues in recent years, the demand for environmentally friendly materials has emerged. Biodegradable polymers made from renewable resources are an alternative solution. This study explores the potential of biocomposite materials made from agricultural wastes and leaves, namely, fungal foams, as a novel renewable resource for packaging applications. Lignocellulosic resources were used as substrates for growing mushrooms because they have a specific composition of hemicelluloses, cellulose, and lignin, which helps in mushroom growing activities. In addition, the substrates were completely derived from components such as lipids, chitins, and polysaccharides [2].

In the past few years, mycelium-based biocomposites have seen steady growth in popularity as an environmentally friendly option. This development is due to the increase in the mushroom market globally, rendering them the second largest species of cultivated mushrooms in the world [3]. Furthermore, various mushroom species in Malaysia, such as *Agaricus*, *Auricularia*, and *Pleurotus*, are widely cultivated [4]. In this study, we selected *Pleurotus* sp., or oyster mushrooms, including *Pleurotus florida* and *Pleurotus citrinopileatus.* The yellow oyster mushroom, also known as *Pleurotus citrinopileatus*, has great commercial potential because it is easy to cultivate. Moreover, the white oyster mushroom (*Pluerotus florida*) has overtaken the production of other types of mushrooms due to their rapid growth and demand requirement [5]. Composites, in their simplest form, are materials comprising two or more elements that significantly vary in their mechanical properties [6]. Mycelium composites are economically advantageous since they are manufactured with low energy and low-cost raw materials or agricultural wastes. Consequently, they are regarded as one of the most promising substitutes for synthetic waste materials such as expanded polystyrenes [7].

Currently, polystyrene and Styrofoam are widely used in various industries, especially for packaging materials, due to their light weight and hard properties. In addition, these polymers can be found everywhere, including food stores and groceries shops. However, owing to its chemical properties, polystyrene is not a biodegradable material, and because its recycling procedure is complicated and expensive, the use of polystyrene results in the production of solid waste disposed of in landfills. Hence, fungal foam has been introduced as a new biodegradable material to replace polystyrene in packaging applications. The mechanical properties of polystyrene in terms of hardness, resilience, and springiness were determined and compared with those of fungal foams.

At present, the circular economy and the sustainability of materials play significant roles in consumer decisions. In contrast, mycelium-based composites use fungal mycelium, which is a network of hyphae that bind to lignocellulosic substrates and produce porous composites. Mycelium is a biocomposite that is fully biodegradable at the end of its life cycle because its primary constituents are natural polymers [8]. Consequently, mycelium technology, which uses fungal-based foams, is another option for replacing conventional industrial packaging [9].

This study promotes new biodegradable materials by utilizing an edible mushroom, namely the *Pleurotus* sp., as a lignocellulosic biomass for further industrial applications, including packaging materials. Therefore, in this context, this research aims to characterize fungal foam of edible mushrooms using different substrates, including rice husk, sugarcane bagasse, teak leaves, and sawdust. Hence, this study investigates the fungal growth performance, mechanical properties, and chemical structure of fungal foam by Fourier-transform infrared spectroscopy (FTIR) and evaluates its morphological structure using scanning electron microscope (SEM) and thermal stability and water absorption tests.

## 2. Materials and Methods

### 2.1. Materials

Agriculture materials used as substrates, including rice husk, sawdust, sugarcane bagasse, and teak leaves, were collected from Perlis, Malaysia. Mycelium (*Pleurotus florida* and *Pleurotus citrinopileatus*) were obtained from Padang Besar, Perlis, Malaysia. Calcium carbonate precipitate powder (CaCO_3_) was purchased from HmbG Chemicals (Malaysia), and rice bran was obtained from a local supplier in Perlis, Malaysia. Distilled water was used consistently throughout the experimental process.

### 2.2. Preparation of Substrates

Substrates for growing mushrooms consisted of lignocellulosic materials, which involved rice husk, sawdust, sugarcane bagasse, and teak leaves. Rice husk and teak leaves were dried in the oven for 12 h at 70 °C before being ground into powder. Sugarcane bagasse was soaked in water and dried under sunlight. This step was repeated for three days before being fully dried in the oven and ground into a powder. As for the sawdust, it could be used immediately after collection from the supplier without drying or grinding.

### 2.3. Production of Fungal Foam Bio-Composites

A medium for growing mushrooms and forming fungal foam was prepared. Substrates, rice bran, and calcium carbonate powder were mixed together in a ratio of 100:10:1, respectively [10]. Each type of substrate was prepared separately in four different basins. After the mixtures were prepared, distilled water was added slowly into the mixture. There was no specific volume of distilled water used; hence, water was added until the medium formed clump-like features, suggesting that the moisture content was adequate. It should be mentioned that different types of substrates require different amounts of water to form a clump. Nonetheless, the substrates were then placed in cups for the growing process, which were subsequently sterilized in an autoclave at 121 °C for 30 min. Under laminar flow, substrates were then inoculated with mycelium spawn before incubation at room temperature. After that, the samples were assessed every three days to analyze the growth performance for every type of mycelium species and substrate. After approximately 30 days, fungal foams were produced, and the foams were immediately placed in a convection oven at 60 °C for 10 h to inactivate the fungus and prevent reanimation and contamination.

### 2.4. Assessment of Morphological Structure

The morphological structure of the fungal foam was determined using an SEM brand model JSM-6390LV (JEOL, Peabody, MA, USA). The fungal foams were cut into small pieces, and samples were sputter-coated with a thin coating of platinum to prevent electrostatic charge during scanning and inspection.

### 2.5. Determination of Mechanical Properties

Texture analysis was run to identify the mechanical properties of the fungal foam using a Stable Micro Systems Texture Analyzer. Three parameters were studied, namely, hardness, springiness, and resilience. Each sample was trimmed to a flat surface before being placed under compression platens connected to the deflect meter. A round surface probe (P/75) was used since fungal foams were produced in a cylindrical shape with a round surface. A double bite technique was implemented in this testing to determine each property mentioned. All testing procedures were performed again on the polystyrene, which was used as the control in the present study.

### 2.6. Chemical Structure Analysis

FTIR spectra were obtained using the KBr method due to the samples being prepared as a solid powder. The range of wavenumbers used was 4000–650 cm^−1^ using PerkinElmer Spectrum Version 10.5.2. Beforehand, pellets of samples were prepared by mixing solid samples and KBr thoroughly while grinding with a pestle. The samples were then placed just enough in the pellet die before being pressed and used to analyze the chemical structure of fungal foams.

### 2.7. Determination of Thermal Properties

The thermal degradation of fungal foams was determined using Thermogravimetric analysis (TGA) Q50 V20.13. The testing was conducted under a supply of nitrogen gas with a flowrate of 50 mL/min. At this flowrate, the nitrogen gas does not interfere with samples during thermal treatment and instead facilitates an inert atmosphere to avoid the oxidation of samples that will affect the collected data. The heating rate of TGA was set to 20 °C/min, and the samples were gradually heated from 30 °C until a maximum of 600 °C. Simultaneously, both the weight and its derivative weight were plotted against temperature.

### 2.8. Water Absorption Test

A water absorption test was carried out on every sample to determine the water uptake of the fungal foam with different mycelium types and different substrates. Therefore, all eight samples were tested using Analytical balance Sartorius types. First, the dried samples were pre-weighted to acquire the initial reading. Then, the samples were soaked in distilled water for 24 h in 16 separated beakers. After 24 h, the samples were removed from the beakers and wiped using laboratory tissue paper to remove extra water on their surface. After 24 h of soaking, the weight was measured and recorded. Finally, water absorption was calculated using Equation (1) [11]

(1) $$\%\;Absorption=\frac{w^{\prime }-w}{w}\times 100$$

w = initial weight of samples
$w^{\prime }$ = weight gained of samples

## 3. Results and Discussion

### 3.1. Fungal Growth Performance

**Figure 1:**
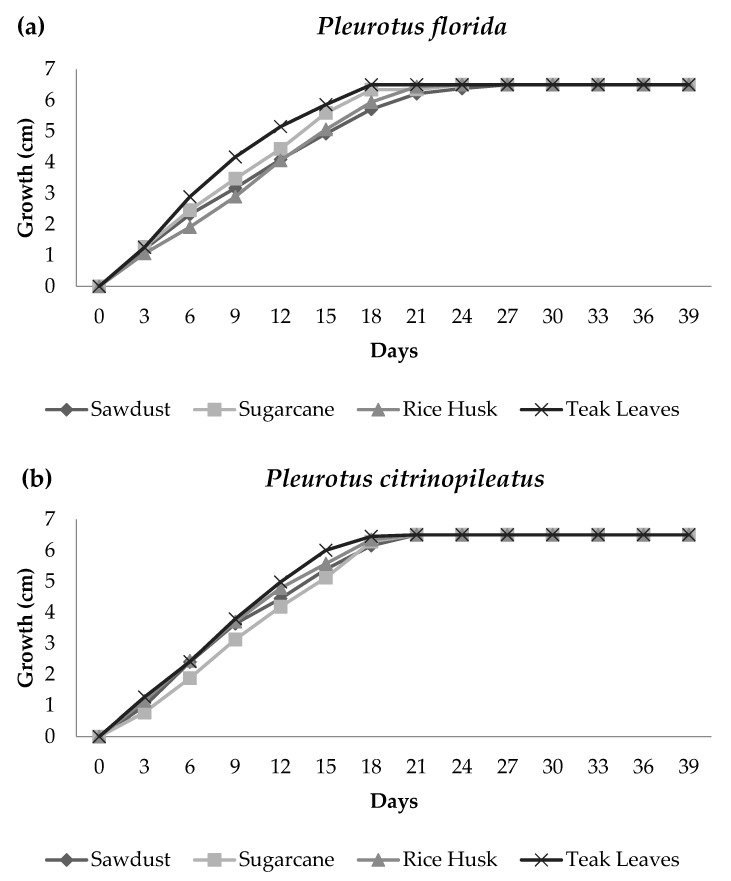
Growth rate of (**a**) *Pleurotus florida* fungal foam (**b**) *Pleurotus citrinopileatus* fungal foam in different substrates.

Figure 1a,b demonstrates the growth performance of the white oyster (*Pleurotus Florida*) and yellow oyster (*Pleurotus citrinopileatus*) mushrooms in four different lignocellulosic substrates in the form of a linear graph. Growth was interpreted at 3-day intervals during foam formation, and the height of the fungal growth was marked on the cup to observe the pattern of the growing phase of both mushroom species. The average fungal growth in both species was highly dependent on the types of substrates used because different species have different preferred substrates. In addition, the activity of enzymes released by *Pleurotus* to absorb nutrients and stimulate mycelium growth is also bound to affect fungal growth [2].

Referring to Figure 1a, there were no significant differences in growth performance between the four substrates used to grow the white oyster mushroom. However, the fungal foam from teak leaves had a slightly higher growth performance compared to the other types, suggesting that teak leaves had better potential for growing this type of mushroom. Fungal foam using teak leaves required only 18 days to fully grow as opposed to sawdust, which required 27 days. Sugarcane and rice husk required the same total number of days for growing fungal foams (24 days).

Figure 1b exhibits the growth rate of the yellow oyster mushroom for which only a slightly different growth rate was noted among all four substrates. The growth differences could only be seen at the beginning (days 1–18), where teak leaves once again showed a better potential to grow the oyster mushroom compared with other substrates. In contrast, mycelium reached full growth at day 21 in all four substrates. In addition, according to the findings from Nashiruddin et al. [2], who utilized rice husk, sugarcane bagasse, and sawdust to cultivate *Pleurotus ostreatus*, the rapid growth of mycelium was found when using a rice husk as a substrate. That research study, however, revealed that teak leaves facilitated a quicker growth of *Pleurotus* sp. and were thus better substrates than the rice husk.

Additionally to that, teak leaves had lower lignin contents compared to rice husk, sawdust, and sugarcane bagasse, which is consistent with the findings of Abdel-Hamid et al. in which *Pleurotus* sp. were found to attack lignin more rapidly than hemicellulose and cellulose. Table 1 shows the lignin content of the substrates [12]. Furthermore, *Pleurotus* sp. required less time to delignify lignin in teak leaves, probably because the lignin level in teak leaves was slightly low. These carbohydrate polymers were depolymerized by the delignification process, leading to the release of free sugars that were crucial for the growth of mycelium since they provide the essential nutrients for fungal growth. Additionally, a decrease in compressive strength was observed as cultivation time increased. This occurred because of the rapid decomposition of a huge quantity of organic substrate [13]. Therefore, it is critical to determine the mechanical properties of the fungal foam.

### 3.2. Mechanical Properties

**Figure 2:**
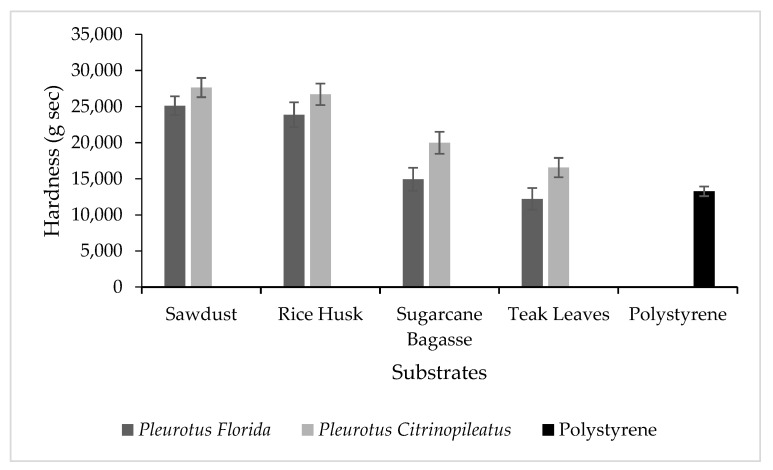
Hardness for different mushroom species of fungal foam in various types of substrates and polystyrene as the control.

Hardness is a measure of a material’s resistance to the application of a force that results in a change to its shape [25]. The compressive strength test is a common way of evaluating biodegradable packaging, and Holt et al. [26] examined this standard test procedure. It is important to carry out compressive strength studies to identify the firmness of the fungal foam, especially since the quality of the foam is indicated by its ability to withstand impacts. Consequently, samples with greater hardness have better impact resistance, rendering them more appropriate as protective materials. Figure 2 shows the hardness of fungal foam produced from different substrates and polystyrene as the control.

Consistent with previous research, this study demonstrated that the compression strength of fungal foam varied according to the type of fungal species and lignocellulosic residue [27,28,29,30]. Sawdust and rice husk showed significantly higher hardness compared to sugarcane bagasse and teak leaves. This result was confirmed in both *Pleurotus florida* and *Pleurotus citrinopileatus* because the structure of the substrates contributed to the hardness of the fungal foam. Rice husk contains hollow tubes in its structure, making it easier for fungi to grow compared to substrates with branches and tight spaces [31,32]. In contrast, even though sawdust showed higher hardness values, it is a type of biomass with a small structure that completely disaggregates grains, exhibiting low material properties. However, utilizing *Pleurotus* sp. mushroom with the mycelium part produced an interior framework, rendering it an excellent substrate. Therefore, the resultant material had very good mechanical properties. Mycelium and sawdust are suggested to produce a compacted material with properties such as the ones shown by polystyrene [33]. Moreover, a previous study by Tacer-Caba et al. [7] showed that the strength of fungal foam or mycelium composites is largely reliant on the substrate used, which also affects the morphology of the fungal foam. In contrast, our data showed that polystyrene hardness was comparable to the fungal foam produced in this study, which, although lower, was still in the same range.

However, to our knowledge, there have not been any previously published reports on the hardness of the fungal foam. As hardness is the ability of a material to resist deformation, this result can be compared with previous studies investigating the compressive strength of fungal foam. More specifically, Tacer-Caba et al. [7] discovered that foam made from *G. lucidum*, which was grown on oat husks and rapeseed cakes, had a higher compressive strength than foam made from *A. bisporus* and *Pleurotus ostreatus*, also grown on the same substrate. In contrast, Bruscato et al. [34] found that *Pleurotus sanguineus* fungal foam grown on pine sawdust exhibited a higher compression strength than *Pleurotus albidus* foam. Finally, Ghazvinian et al. [13] reported that the compressive strength of *Pleurotus ostreatus* fungal foam grown on sawdust (1.02 MPa) was greater than that grown on straw (0.07 MPa).

**Figure 3:**
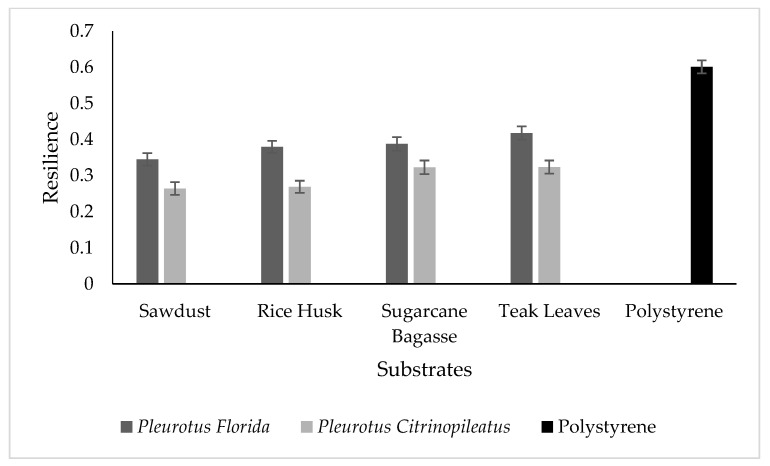
Resilience for different mushroom species of fungal foam in various types of substrates and polystyrene as the control.

Figure 3 shows the resilience of fungal foam using various agriculture wastes and leaves as substrates and also polystyrene. In general, resilience reflects the ability of fungal foam to regain its original position after impact and withstand shock, absorb energy, and impact loads [33]. This criterion is essential and exhibits the potential of fungal foam to be used as a protective packaging material that is not easily broken. As stated by Islam et al. [35], the density of fungal mycelium has a significant impact on the stress–strain relationship under compression in fungal mycelium. When the tension is removed, fungi have the ability to “fight” to regain their original position due to hyphal branching, which generates a random fiber network structure. Teak leaves and sugarcane bagasse have more compact structures, as indicated by morphological tests, which contributes to increased resilience because their fungal hyphae network aids in regaining their original state. This might in turn explain why teak leaves and sugarcane bagasse-producing fungal foams had higher resilience compared to other fungal foams, although slightly lower than that of polystyrene.

**Figure 4:**
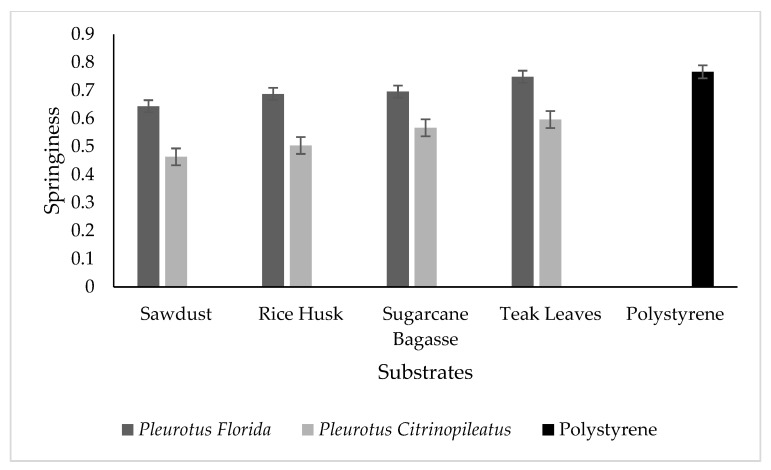
Springiness for different mushroom species of fungal foam in various types of substrates and polystyrene as the control.

Springiness or elasticity is a flexible characteristic of fungal foam and reflects its ability to return to its original size and shape after a force is removed. The graph plotted in Figure 4 demonstrates that there is no significant difference in springiness between the types of substrates used. However, the mycelium species showed a slightly different value, for which the white oyster mushroom had higher springiness compared to the yellow oyster. When the value of springiness approaches 1, the products are indicated to be more elastic. As the product formed in the present study is fungal foam from mushrooms, the springiness of the samples is higher because mycelium has its own elastic properties. In addition, this result confirmed the potential of fungal foam to be utilized as a packaging material as the springiness value is comparable to the polystyrene that is currently used in industry. Overall, springiness is the property of being springy, whereas resilience is the energy absorbed by a material to regain its original shape. However, there is no previous research focusing on the springiness and resilience of fungal foam. Most of the previous studies have investigated the elasticity of fungal foam or mycelium-based composites.

### 3.3. Morphological Analysis

**Figure 5:**
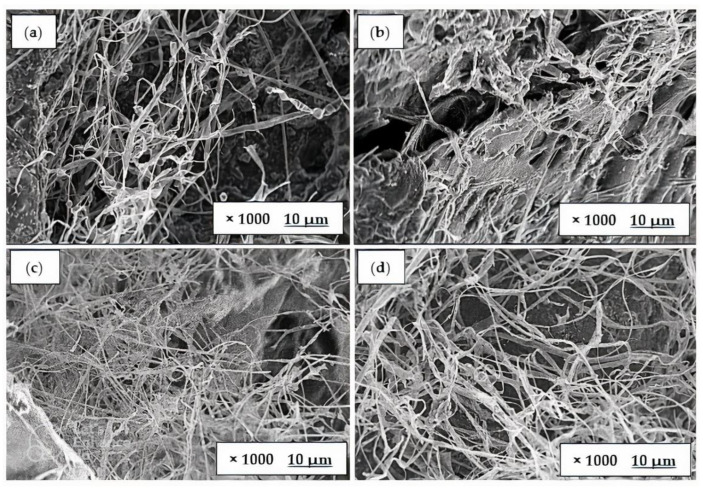
Morphology of fungal foam using *Pleurotus florida* in (**a**) rice husk, (**b**) sawdust, (**c**) sugarcane bagasse, and (**d**) teak leaves.

**Figure 6:**
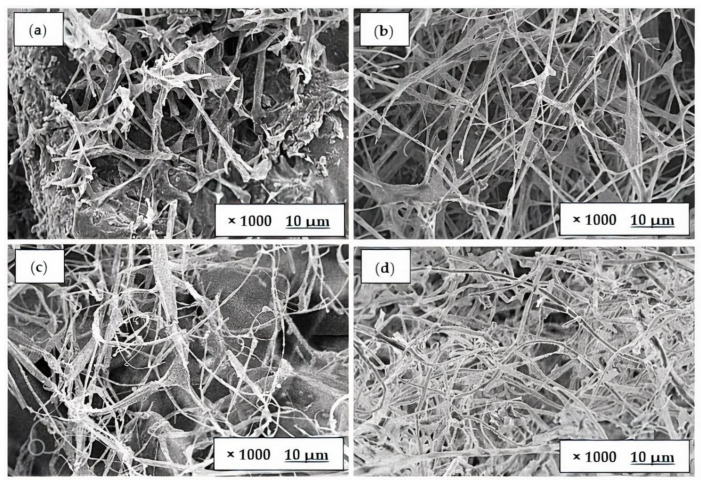
Morphology of fungal foam using *Pleurotus citrinopileatus* in (**a**) rice husk, (**b**) sawdust, (**c**) sugarcane bagasse, and (**d**) teak leaves.

Figure 5 and Figure 6 depict the morphology of fungal foam from white and yellow oyster mushrooms, respectively, grown in different lignocellulosic biomasses as substrates. A mycelial film covered the surface of all materials; however, the texture and porosity of the membrane varied according to the type of substance used [36]. Both Figure 5 and Figure 6 demonstrate that the hyphae of fungal foam in sugarcane bagasse (c) and teak leaves (d) is more compact and has higher mycelium networks compared with the fungal foam in rice husk (a) and sawdust (b). The mechanical property evaluation revealed a contradictory correlation between hardness and compact filaments, indicating that the mycelium filaments present in the fungal foam contributed to this characteristic.

Moreover, mycelium grown in all substrates showed that hyphae structures adhered to the substrates used rather than to each other, which could be explained by the availability of nutrients on substrates. This result was also supported by Bruscato et al. [34], who used sawdust to grow *Pleurotus Albidus*, and the foam’s morphology revealed that compact filaments consisted of many hyphae that stuck to the substrates rather than to each other. In regard to the mycelial component itself, Haneef et al. [37] found that the substrate had a significant impact on the mycelial composition of polysaccharides, lipids, and chitin, and on the overall morphology and mechanical properties. In comparison to its composition, mycelium showed unexpected and seemingly contradictory properties. Specifically, *Pleurotus ostreatus* was found to be stiffer than *G. lucidum*, which was believed to be related to its higher polysaccharide content.

### 3.4. FTIR Analysis

**Figure 7:**
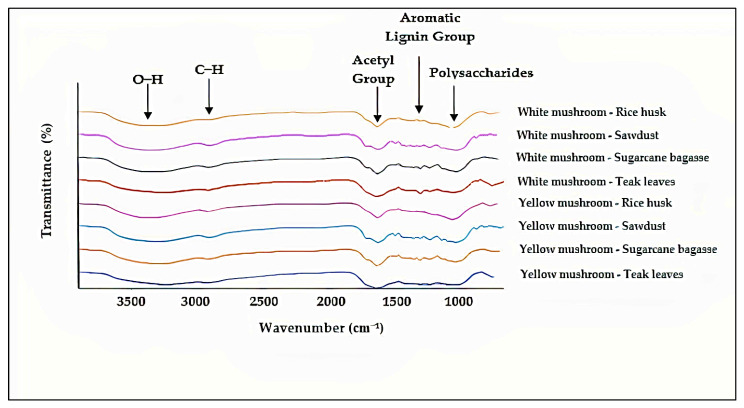
FTIR spectra of fungal foam from *Pleurotus florida* and *Pleurotus citrinopileatus* in different substrates.

FTIR analysis was performed between the 650 and 4000 cm^−1^ wave number region. Figure 7 shows the FTIR spectra of the white oyster mushroom and yellow oyster mushroom utilizing rice husk, sugarcane bagasse, teak leaves, and sawdust as substrates. The infrared absorption spectra of the fungal foams are associated with the molecules present in the mycelium and substrate media [34], e.g., polysaccharides (1200–900 cm^−1^), nucleic acids (1255–1245 cm^−1^), proteins (amide I at 1700–1600 cm^−1^, amide II and III at 1575–1300 cm^−1^), and 234 lipids (3000–2800 cm^−1^, ~1740 cm^−1^) [37].

The stretching vibration of the O–H groups in cellulose and hemicellulose was identified as the source of the peak at 3379–3252 cm^−1^. According to Peng et al. [36], the stretching vibration of the O–H group in the remaining moisture of the samples, cellulose, and hemicellulose causes a broad band at 3500–3000 cm^−1^ in all composites. Next, the peak at 2925–2919 cm^−1^ was found to be the result of the stretching vibration of the C–H groups in waxes and oils. Peaks between 1728 and 1644 cm^−1^ were identified as the carbonyl stretching vibration of the acetyl group in hemicelluloses and methyl ester. Furthermore, the stretching vibration of aromatic rings in lignin was responsible for the peaks at 1375–1323 cm^−1^. Peaks at 1107–1056 cm^−1^ were attributed to C–O, C–C, and C–O–C stretching vibrations in polysaccharides.

### 3.5. Thermogravimetric Analysis

**Figure 8:**
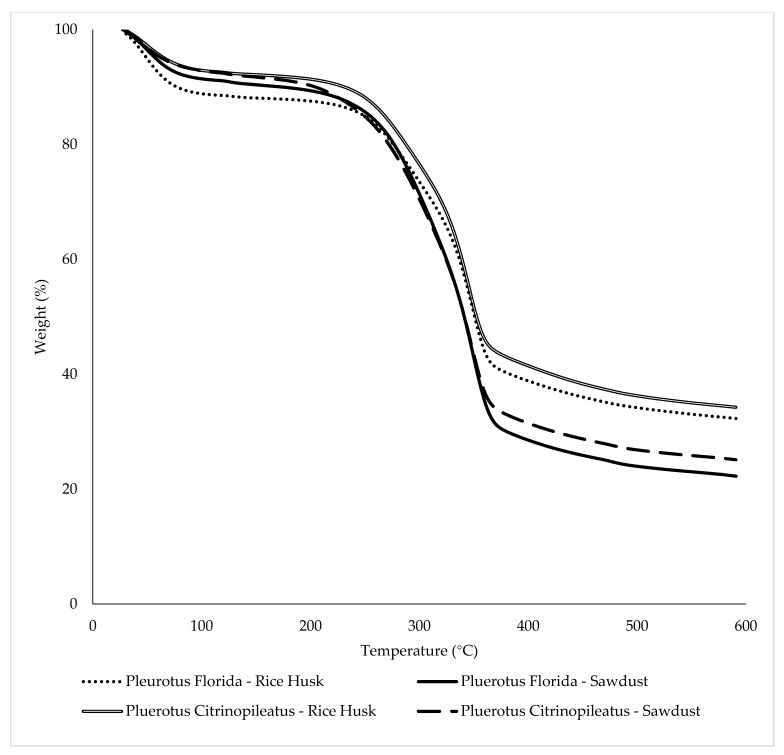
Thermogram of fungal foam from *Pleurotus florida* and *Pleurotus citrinopileatus* with varying substrates.

The thermal stability of fungal foams of *Pleurotus Florida* and *Pleurotus citrinopileatus* using rice husk and sawdust were evaluated by Thermogravimetric (TGA) with temperatures ranging between 30 °C and 600 °C, as shown in Figure 8. There were three main phases in this test, namely, the drying and evaporation stage, the devolatilization stage, and the decomposition stage [38].

The first phase occurred between 40 °C and 130 °C. In this stage, the white oyster mushroom in rice husk showed the highest percentage of weight loss, which was 11.04%, followed by the white oyster mushroom in sawdust (8.46%). As for the yellow oyster mushroom, both rice husk and sawdust had less than 8% weight loss. The next stage of weight loss occurred at temperatures between 200 °C and 450 °C, where the devolatilization of the biocomposites takes place. There is a specific phase in the breakdown of lignocellulosic materials such as cellulose, hemicellulose, and lignin [34]. However, there were no significant differences between the thermal degradation of hemicellulose and cellulose at that stage. In addition, all samples showed a similar trend to the thermal temperature degradation of cellulose because the cellulose composition of substrates does not differ significantly among the substrates (50% in rice husk [39], 36% in sugarcane bagasse [22], 36.9% in teak leaves [40], and 41.58% in sawdust [16]). The third phase involved the decomposition of lignin at temperatures over 500 °C. At this stage, the degradation of the primary residual char resulted in the production of carbonaceous char residue until the burning sample was stopped at 600 °C [41].

**Figure 9:**
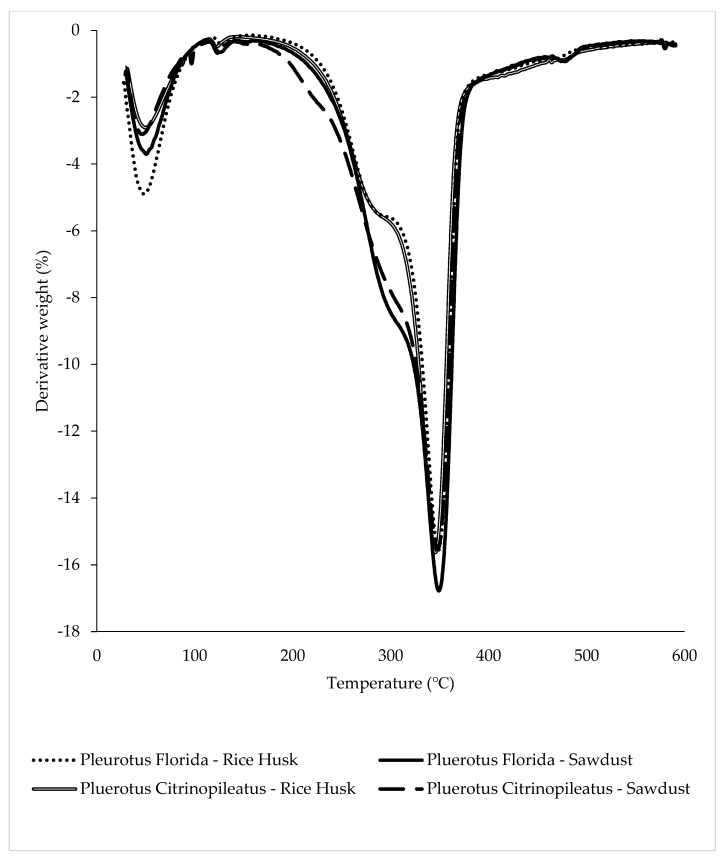
Differential thermogravimetry (DTG) curve of fungal foam from *Pleurotus florida* and *Pleurotus citrinopileatus* with varying substrates.

Figure 9 shows the peak of the maximum temperature, which represents the char residual degradation temperature, for *Pluerotus Florida* (350 °C) and *Pluerotus Citrinopileatus* (375 °C). As demonstrated by the degradation peak of the DTG curve, the fungal foam from the yellow oyster mushroom was more resistant to heat than the fungal foam from the white oyster mushroom regardless of the substrate used. The yellow oyster mushroom exhibited a more stable thermal degradation of the char material compared to the white oyster mushroom.

### 3.6. Water Absorption of Fungal Foam

**Figure 10:**
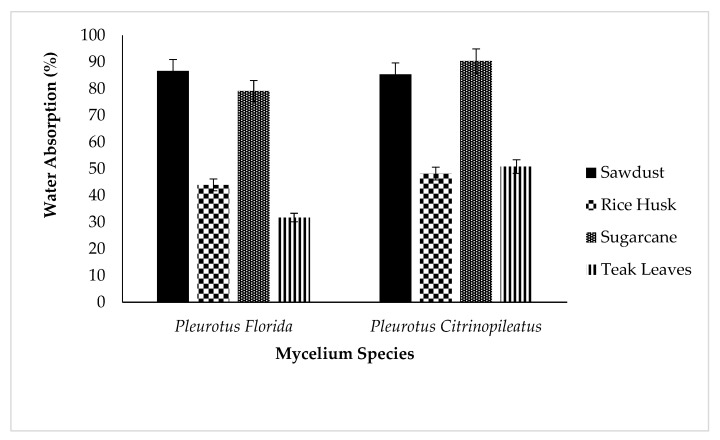
Water absorption of fungal foam from *Pleurotus florida* and *Pleurotus citrinopileatus* in different substrates.

We then evaluated the water absorption ability of fungal foams to determine the most appropriate samples that could be used for packaging materials. Usually, the best fungal foams absorb the lowest percentage of water after immersion for 24 h. Figure 10 demonstrates that the water absorption of fungal foams using rice husks and teak leaves as substrates was 44–48% and 32–50%, respectively. In contrast, *Pleurotus florida* grown in teak leaves as the substrate absorbed 31.74% of water after 24 h. This result is consistent with the findings of Elsacker et al. [30] who used mycelium species of *Trametes versicolor* grown in chopped flax as substrates and showed water absorption of 30.28% after 24 h. Moreover, this result can be explained by the hydrophobic characteristics of various substrates. Rice husk consists of 15–20% of silica [39], and the hydrophobic behavior of silica results in the low water absorption and permeability of rice husk [42].

In addition, the amount of water absorption was influenced by the chemical composition of the substrates. Hemicellulose is responsible for a considerable amount of water absorption. Sawdust and sugarcane bagasse are characterized by a significantly higher hemicellulose content compared to rice husk and teak leaves, as shown in Table 2, which subsequently explains why fungal foam produced from sugarcane bagasse and sawdust absorbs more water. Hemicelluloses are found mostly in primary cell walls and have a complex chemical structure with several branches. There are both 5- and 6-carbon ring sugars in hemicelluloses, and cellulose microfibrils are held together by a matrix of hemicelluloses. Hemicelluloses are highly hydrophilic, and the acid groups in hemicelluloses are primarily responsible for their high hydrophilicity, which increases their capacity to absorb water within their fibers [43]. Additionally, due to the high-water absorption rates, fungal foams from sawdust (85–86%) and sugarcane (80–90%) are unsuitable for application in the waterproof packaging material industry, and thus need to be modified. Due to its high water-absorbing capacity, fungal foam can degrade more quickly, increasing its biodegradability. The fungal foam becomes more favorable to the growth of degrading microorganisms as more water is absorbed [44].

## 4. Conclusions

The fungal foam with teak leaves as the substrate indicated the fastest growth rate (18 days) due to the lower lignin content. On the contrary, the other substrates required 24–27 days to fully grow. With respect to its mechanical properties, the fungal foam from sawdust was the hardest material, and *Pleurotus citrinopileatus* was significantly harder than *Pleurotus florida*. In TGA analysis, *Pleurotus citrinopileatus* exhibited higher resistance to heat compared to *Pleurotus florida*. For both types of mushrooms, water absorption tests revealed that fungal foams made from rice husk and teak leaves absorbed less water compared to other substrates. For *Pleurotus florida*, the fungal foam made from sawdust exhibited higher water absorption (273%) than teak leaves, whereas fungal foam made from sugarcane bagasse demonstrated higher water absorption (187%) than rice husk for *Pleurotus citrinopileatus.* In brief, fungal foams made from oyster mushrooms by inoculating mycelium species in agricultural byproducts and leaves have great potential to be used as packaging materials.

## Figures and Tables

**Table 1:** Lignin content of the substrates used.

Substrate	Lignin Content	References
Teak leaves	19–20%	[14,15]
Sawdust	24–34%	[16,17,18]
Rice husk	21–29%	[19,20,21]
Sugarcane bagasse	21–24%	[22,23,24]

**Table 2:** Hemicellulose content of different substrates.

Substrate	Hemicellulose Content	References
Teak leaves	19–26%	[14,39]
Sawdust	27–33%	[16,17,18]
Rice Husk	15–20%	[19,20,21]
Sugarcane bagasse	30–39%	[20,21,22,23,24]

## Data Availability

The data presented in this study are available on request from the corresponding author.
